# Heart failure due to cytomegalovirus myocarditis in immunocompetent young adults: a case report

**DOI:** 10.1186/s13104-016-2181-5

**Published:** 2016-08-05

**Authors:** Moacyr Magno Palmeira, Hellen Yuki Umemura Ribeiro, Yan Garcia Lira, Fernando Octávio Machado Jucá Neto, Ivone Aline da Silva Rodrigues, Letícia Nazareth Fernandes da Paz, Maria da Conceição Nascimento Pinheiro

**Affiliations:** 1 Section of Urgency and Emergency, Division of Cardiology, Department of Intensive Care, Fundação Pública Estadual Hospital de Clínicas Gaspar Vianna, 2000 Alferes Costa street, Pedreira, 66087-660 Belém, PA Brazil; 2 Section of Urgency and Emergency, Division of Cardiology, Department of Clinical Care, Universidade do Estado do Pará, 2623 Perebebuí street, Marco, 66087-670 Belém, PA Brazil; 3 Section of Human Pathology, Division of Tropical Diseases, Department of Infectious Diseases, Universidade Federal do Pará, 92 Generalíssimo Deodoro avenue, 66055-240 Umarizal, PA Brazil; 4 60 Amazonas square, Jurunas, 66025-070 Belém, PA Brazil

**Keywords:** Cytomegalovirus infection, Myocarditis, Heart disease

## Abstract

**Background:**

Cardiac complications constitute a rare clinical manifestation of cytomegalovirus (CMV) infection. This virus is usually asymptomatic in immunocompetent individuals. We report a case of myocarditis and cardiac insufficiency due to primary CMV infection. Serological tests by using ELISA method showed positive results for the virus.

**Case presentation:**

A 41-year-old man with no prior comorbidities presenting with dyspnoea, fever, and oedema was admitted to the cardiac emergency service. He had fever and dry cough, which aggravated into progressive respiratory distress, lower limb oedema, and orthopnoea 30 days prior to hospitalisation. The electrocardiogram revealed sinus tachycardia, first-degree right bundle branch block, and ventricular and left atrial overload as well as diffuse and nonspecific disturbances of ventricular repolarization. Serological tests were conducted, and IgM (1.54 UI/mL) and IgG (2.5 UI/mL) were found positive only for CMV by using ELISA. The patient was diagnosed with cardiac insufficiency due to CMV myocarditis. He was treated with ganciclovir for 10 days and received supportive medication.

**Conclusion:**

This case reaffirms the possibility of cardiac involvement in CMV infection and emphasises the importance of viral aetiologies as differential diagnoses for acute myocarditis.

## Background

Cytomegalovirus (CMV) infection is a prevalent infectious disease worldwide; however, its prevalence varies with geographical regions and socioeconomic statuses [1]. CMV belongs to the Herpesviridae family and Betaherpesvirinae subfamily type 5; it is a DNA virus with the ability of remaining latent for a long time [2]. Its prevalence ranges from 30 to 100 % in the general population with prior exposure to the virus as determined by serological tests. This prevalence is inversely proportional to socioeconomic status and directly proportional to age [3]. Adults and adolescents, such as day-care workers and school teachers, are at higher risk for acute CMV infection because of the close contact with children who may be infected [1]. Primary infection is usually an asymptomatic event in immunocompetent individuals. However, there are clinical presentations that are described as classic influenza-like syndrome or mononucleosis-like syndrome with symptoms of the classic fever, pharyngitis, and lymphadenopathy triad [1, 5]. The intensity and clinical symptoms vary and include systemic disorders such as hepatitis, hematologic diseases, or meningoencephalitis; additionally, it can be associated with high mortality rates [2, 4, 5].

Cardiovascular complications constitute a rare manifestation of CMV infection; it is a diagnostic challenge in the emergency cases [4]. We describe a previously healthy man who developed myocarditis and cardiac insufficiency due to primary CMV infection.

## Case presentation

A 41-year-old man, unemployed, brown, parent of two school-age children, with no prior comorbidities, and presenting with dyspnoea, fever, and oedema was admitted to the cardiac emergency service. The patient had undergone no related interventions in the past and had no cardiac risks based on his family history.

**Fig. 1:**
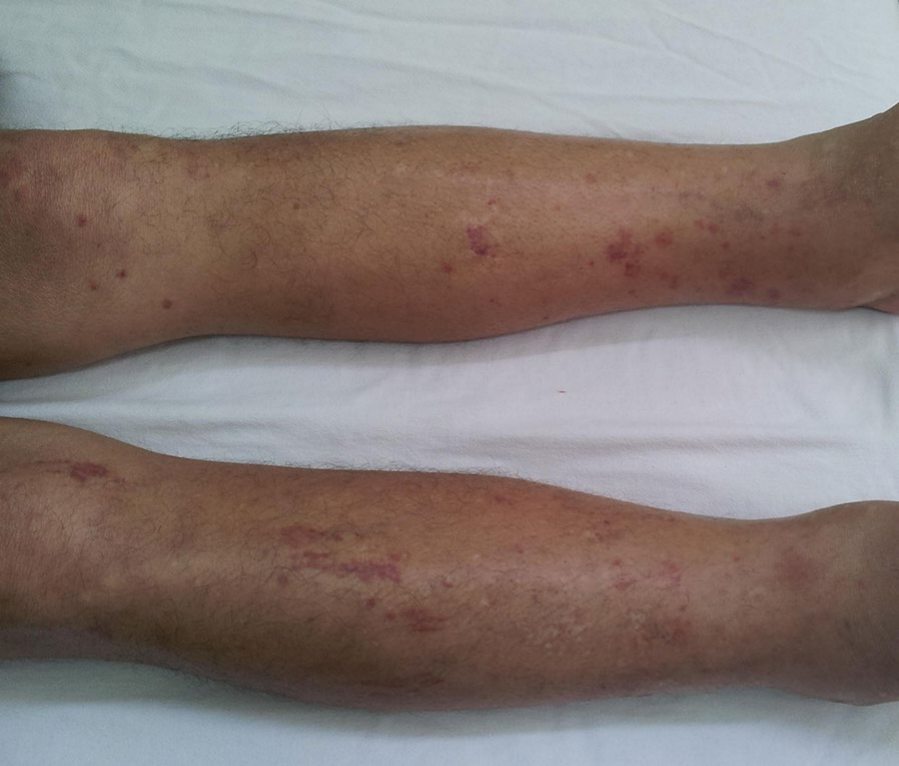
Patient’s lower limbs presenting with severe oedema, ecchymosis, and petechiae. Patient presented with painless oedema, positive pitting oedema (++++/4+) of the lower limbs, ecchymosis, and petechiae

He had fever and dry cough, which aggravated into progressive respiratory distress, lower limb oedema, and orthopnoea 30 days prior to hospitalisation. Physical examination revealed fever (38 °C), pallor (++/4+), dyspnoea, tachycardia [heart rate (HR) = 136 bpm], cardiac gallop rhythm (presence of S3), mitral systolic murmur as well as absent breath sounds at the lung base, and crackles and rales up to the middle third of both the lungs. Additionally, he experienced hepatomegaly, splenomegaly, painless oedema, positive pitting oedema (++++/4+) of the lower limbs, bruises, and petechiae (Fig. 1).

**Fig. 2:**
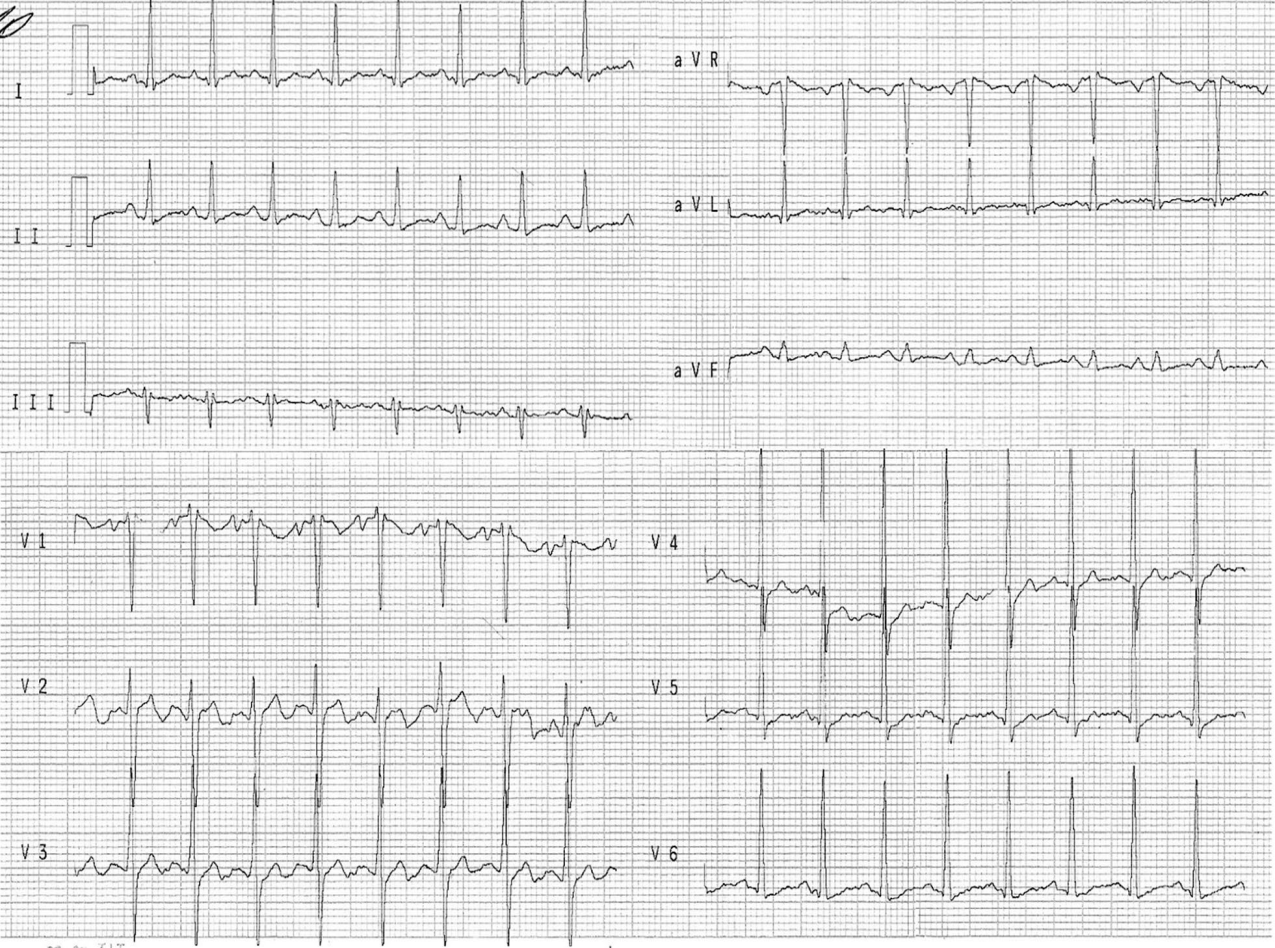
Electrocardiogram revealing sinus tachycardia, first-degree right bundle branch block, and other cardiovascular disorders. The electrocardiogram reveals sinus tachycardia, first-degree right bundle branch block, and ventricular and left atrial overload, as well as diffuse and nonspecific disturbances of ventricular repolarization

**Fig. 3:**
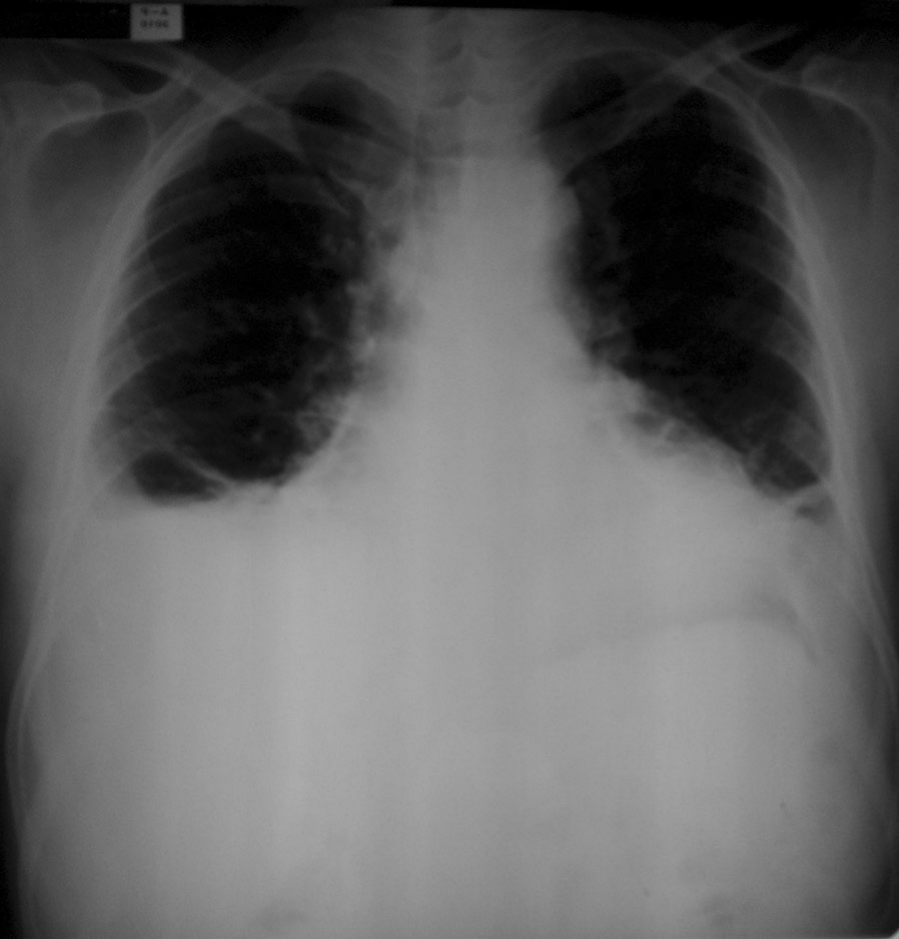
Chest radiograph showing cardiomegaly and bilateral pleural effusions in the lung bases. The chest radiographs reveal bilateral pleural effusion in the lung base and an enlarged cardiac silhouette

The electrocardiogram revealed sinus tachycardia, first-degree right bundle branch block, and ventricular and left atrial overload as well as diffuse and nonspecific disturbances of ventricular repolarization (Fig. 2). The chest radiographs revealed bilateral pleural effusion in the lung base and an enlarged cardiac silhouette (Fig. 3). A transthoracic echocardiogram revealed 35 % ejection fraction (EF) of the left ventricle (LV) and an increase in the left cavity (70 mm LV). Coronary angiography revealed no obstructive atherosclerosis lesions in the coronary artery.

Serological tests were conducted for HIV-1 and HIV-2, hepatitis B and C, CMV, and Chagas disease. The serological tests results were negative for all the viruses except CMV, which included positive results for IgM (1.54 UI/mL) and IgG (2.5 UI/mL) by using ELISA.

Laboratory-based tests conducted upon admission revealed the following: haemoglobin level, 9.15 g/dL; leukocyte number, 8400/mm^3^; segmented neutrophil fraction, 77.5 %; platelet number, 289,000/mm^3^; unchanged coagulation; erythrocyte sedimentation rate, 120 mm/h; and C-reactive protein (CRP) level, 96 mg/dL. Troponin I level increased but the creatinine kinase-MB level did not increase (3.12 and 2.1 ng/mL, respectively). The renal scores and ionogram values were within the reference values. In addition, the patient showed significantly increased levels of canalicular liver enzymes [alkaline phosphatase (ALP) and gamma glutamyl transferase (GGT)] during hospitalisation.

**Fig. 4:**
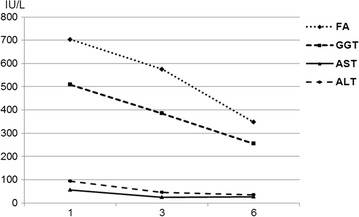
Laboratory tests-based evolution of liver enzymes during ganciclovir therapy. After receiving the anti-viral medication, the patient exhibited a regression in his liver enzyme levels based on laboratory tests; changes are shown from day 1 to day 6 of the therapy

The patient was diagnosed with cardiac insufficiency due to CMV myocarditis. He was administered 250 mg of ganciclovir IV twice a day for 10 days and received supportive medication that included oral administration of 0.25 mg of digoxin once a day, 50 mg of losartan twice a day, 25 mg of carvedilol twice a day, 40 mg of furosemide twice a day, and 25 mg of spironolactone once a day. After receiving the anti-viral medication, the patient’s liver enzyme levels decreased, as revealed by laboratory-based tests (Fig. 4); in addition, the hepatosplenomegaly improved. Abdominal CT was performed following the drug therapy, and normal liver characteristics were revealed with no biliary tract dilatation. The ecchymotic spots and petechiae that were observed upon admission and that had emerged during the course of infection were also treated.

The patient showed improvement in clinical and laboratory-based tests and was discharged in functional class I (New York Heart Association) after 14 days of hospitalisation. The serological tests confirmed CMV infection (IgM, 0.9 UI/mL and IgG, 10.1 UI/mL). One month after discharge, routine echocardiography revealed a 75 % EF of the LV and almost normal values for the diameter of the left chambers. The cardiac enzyme levels were normal.

## Discussion

Generally, in immunocompetent adults, CMV infection is either asymptomatic or involves a typical disease course similar to a self-limited mononucleosis-like syndrome. The infection rarely causes severe complications of specific organs; however, acute gastrointestinal, cardiovascular, neurological, and/or liver disorders have been reported [1].

Evidence of cardiac events, particularly acute pericarditis or myocarditis due to CMV infection, is uncommon in immunocompetent patients without any associated risk factor [4]. Normally, these changes occur asymptomatically and, in most cases, are diagnosed as incidental findings during the typical course of mononucleosis-like syndrome [4, 5]. However, there may occasionally be serious complications associated with infection, such as cardiogenic shock, tamponed, or acute cardiac insufficiency [5].

The diagnosis of myocarditis is a constant challenge in medical practice because of high clinical variability and high risk of sudden death, which can progress to dilated cardiomyopathy in approximately 10 % of these patients [6]. Myocarditis is defined as inflammation of the heart muscle and may involve the myocytes, interstitium, vascular elements, and pericardium [6]. Infectious agents, especially viral agents, are the most important etiologic agents [7].

Owing to their risks and high costs, use of endomyocardial biopsies for making diagnoses remains controversial. The incremental diagnostic, prognostic, and therapeutic values of risks associated with such an invasive procedure must be evaluated. Particularly, such an evaluation must be conducted when the aetiology of myocarditis, based on positive results of CMV serological tests, is very strongly suspected. An excellent clinical outcome after ganciclovir therapy that included >100 % EF increase confirmed the initial diagnosis made by using a non-invasive test [8].

Enteroviruses are widely associated with myocarditis and dilated cardiomyopathy, and CMV has sporadically been implicated in this process. In a survey of etiologic agents in cases of ventricular dysfunction designated as “idiopathic,” expression of the CMV genome was detected in only 0.8 % of the cases [7].

In a study based on myocardial autopsy, the CMV DNA was found in 38 % of the patients with myocarditis as the death cause; in contrast, no CMV DNA was found in the control subjects. The authors suggested that an anti-viral therapy could be potentially effective for treating CMV infection in patients with severe acute myocarditis; our study findings are similar to that of this study. We observed an excellent clinical outcome after ganciclovir therapy [9].

The pathophysiology of viral myocarditis involves a complex process characterized by three distinct phases: infection of myocytes, production of toxins, and immune-mediated cytotoxicity. Thus, injury occurs because of the direct cytotoxic effect of the causative agent, the secondary immune response caused by the infectious agent, cytokine expression in the myocardium, and the aberrant induction of apoptosis. It is believed that the progression to dilation and contractile dysfunction occurs in a linear manner and that the manifestation of symptoms may occur at any of the three phases [6].

The prognosis appears to be favourable in patients who survive the initial critical phase. Certain studies have shown a 50–80 % chance of resolving dilated cardiomyopathy within the first 2 years of the clinical onset [6]. In the present case, satisfactory progress was seen that included the recovery of ventricular function and decreased dilation of the cardiac chambers.

Abnormalities based on liver function tests are often reported in immunocompetent adults with clinically significant primary CMV infection. The blood levels of transaminases frequently increase slightly or moderately; however, the levels rarely exceed five times the reference values; in addition, the alanine transaminase (ALT) levels are more affected than the aspartate transaminase (AST) levels are. Increase in ALP, GGT, and total bilirubin levels are uncommon [1].

In this case, minimal change was detected in the AST and ALT levels, whereas the GGT and ALP levels increased significantly, which was expected during viral infection. These findings led to a hypothesis of asymptomatic viral cholangitis with a self-limited course due to anti-viral therapy. These enzymes decreased during therapy, and the abdominal ultrasound examination performed during the patient follow-up revealed no signs of gallstones or liver changes.

Additionally, petechiae and ecchymotic spots were observed on the lower limbs, which had appeared during the course of infection and disappeared a few days after the completion of the drug therapy. This trend suggested the possible evolution of infectious purpura fulminans caused by CMV infection. Vascular involvement by CMV has been reported; this virus induces vascular changes and triggers a cascade of events that can generate perivascular inflammation and even thrombosis [10].

The role of anti-viral therapy in the management of visceral diseases caused by CMV in immunocompromised patients, particularly transplant recipients, has been well established. However, the use of these agents in immunocompetent individuals is not well established, and no definitive conclusion has been established regarding their indication [4]. Nevertheless, several studies indicate the benefits of using anti-viral therapy, particularly during the acute phase of viral infection and in severe cases [6].

In the present case, the acute nature of the viral infection and the severity of the clinical situation prompted us specifically to provide ganciclovir therapy. The outcome was favourable and included the resolution of symptoms and a marked improvement of the cardiac, respiratory, and liver manifestations after specific anti-viral therapy.

## Conclusions

This case reaffirms the possibility of cardiac involvement in CMV infection and emphasises the importance of viral aetiologies as differential diagnoses for acute myocarditis.

## Abbreviations

ALP: alkaline phosphatase
ALT: alanine transaminase
AST: aspartate transaminase
CMV: cytomegalovirus
CRP: C-reactive protein
CT: computerized tomography
EF: ejection fraction
ELISA: enzyme-linked immunosorbent assays
GGT: gamma glutamyl transferase
HIV: human immunodeficiency virus
HR: heart rate
IV: intravenous
LV: left ventricle
